# A Deep Learning Model System for Diagnosis and Management of Adnexal Masses

**DOI:** 10.3390/cancers14215291

**Published:** 2022-10-27

**Authors:** Jianan Li, Yixin Chen, Minyu Zhang, Peifang Zhang, Kunlun He, Fengqin Yan, Jingbo Li, Hong Xu, Daniel Burkhoff, Yukun Luo, Longxia Wang, Qiuyang Li

**Affiliations:** 1 Department of Ultrasound, The First Center of Chinese PLA General Hospital, Beijing 100853, China; 2 Machine Learning Department, BioMind Technology, Zhongguancun Medical Engineering Center, Beijing 100872, China; 3 Department of Ultrasound, The 7th Center of Chinese PLA General Hospital, Beijing 100700, China; 4 Beijing Key Laboratory for Precision Medicine of Chronic Heart Failure and Key Laboratory of Ministry of Industry and Information Technology of Biomedical Engineering and Translational Medicine, Medical Big Data Research Center, Chinese PLA General Hospital, Beijing 100853, China; 5 Department of Ultrasound, Hengshui People’s Hospital, Hengshui 053000, China; 6 Heart Failure, Hemodynamics and MCS Research, Clinical Trials Center, Cardiovascular Research Foundation, New York, NY 10019, USA

**Keywords:** deep learning, adnexal masses, borderline, pathological subtypes

## Abstract

**Simple Summary:**

This was a multicenter study on the development of a deep learning (DL) model system to diagnose adnexal masses on ultrasound images. There were three innovation points. First, the DL system contained five models: a detector, a mass segmentor, a papillary segmentor, a type classifier, and a pathological subtype classifier. Therefore, the system could finish the entire diagnosis process for adnexal masses on ultrasound images. Second, the DL system could discriminate borderline tumors from benign and malignant tumors with the assistance of annotations for papillary projections (which is a significant morphological feature of borderline tumors). Third, the benign tumors were classified into five pathological subtypes with different risks of clinical complication and accurate disease.

**Abstract:**

Appropriate clinical management of adnexal masses requires a detailed diagnosis. We retrospectively collected ultrasound images of 1559 cases from the first Center of Chinese PLA General Hospital and developed a fully automatic deep learning (DL) model system to diagnose adnexal masses. The DL system contained five models: a detector, a mass segmentor, a papillary segmentor, a type classifier, and a pathological subtype classifier. To test the DL system, 462 cases from another two hospitals were recruited. The DL system identified benign, borderline, and malignant tumors with macro-F1 scores that varied from 0.684 to 0.791, a benefit to preventing both delayed and overextensive treatment. The macro-F1 scores of the pathological subtype classifier to categorize the benign masses varied from 0.714 to 0.831. The detailed classification can inform clinicians of the corresponding complications of each pathological subtype of benign tumors. The distinguishment between borderline and malignant tumors and inflammation from other subtypes of benign tumors need further study. The accuracy and sensitivity of the DL system were comparable to that of the expert and intermediate sonographers and exceeded that of the junior sonographer.

## 1. Introduction

Adnexal masses are widespread in women. Most masses are benign and some may disappear spontaneously [1]. Conservative management is often advised [2]. However, some adnexal masses classified as borderline or malignant may pose a significant risk. Ovarian cancer is the most aggressive of gynecological malignancies; radical surgery and adjuvant chemotherapy are recommended according to stage [3,4]. Borderline tumors present no destructive infiltrative growth and fertility-sparing surgery is appropriate [3,5]. According to the International Federation of Gynecology and Obstetrics (FIGO), there are varied pathological origins of adnexal masses [4]. Different pathological subtypes of benign adnexal masses are associated with different complication risks [5,6]. Endometriomas affect fertility and may cause pelvic pain [7]. Dermoid cysts have the risk of adnexal torsion [8]. Sex cord-stromal tumors may present with hormonal change symptoms [9]. Patients with inflammation might require anti-inflammatory treatment to relieve symptoms such as abdominal pain. In order to monitor corresponding complications during observation and perform intervention timely, accurate pathological subtype classification is very important for appropriate management.

Serum tumor markers can be used to detect the presence and risk of ovarian cancer, but they have limited utility in patients with early-stage disease and premenopausal women [10,11]. Due to the risk of disseminating malignant cells, preoperative biopsies are generally not performed [12]. Therefore, the primary diagnosis depends mainly on imaging techniques. MRI provides the best means of evaluating ovarian masses but has low sensitivity for detection of masses [2]. In contrast, ultrasound is considered to be the first-line imaging modality to assess adnexal tumors [13]. To facilitate accurate diagnoses, many pattern-based approaches and logistical models based on image characteristics have been proposed for evaluating adnexal masses [3]. The pattern-based approaches include the simple ultrasound-based rules (SR), the gynecologic imaging reporting and data system (GI-RADS), and the three-step strategy [6,13,14,15]. In addition, extensive databases have been used to develop and validate logistical models such as the logistic regression (LR) model and the assessment of different neoplasias in the adnexa (ADNEX) model [16,17,18,19]. Of these various methods, subjective assessment (SA) by expert sonographers remains the most accurate ultrasound imaging diagnostic approach to distinguish between benign and malignant adnexal tumors, but there is a shortage of expert sonographers in clinical work [3]. Moreover, each of these approaches performs well in different clinical settings (AUC 0.92–0.94) but has limitations when assessing borderline tumors or pathologic subtypes of benign masses [20].

Deep learning (DL) is a state-of-the-art artificial intelligence (AI) technique for automated image analysis [21]. DL can detect subtle features beyond the ability of the human eye, thus enhancing the ability to diagnose and classify diseases from diagnostic images, as already demonstrated in the fields of diabetic retinopathy; echocardiography; fetal heart disease; and skin, thyroid, and gastric cancers [22,23,24]. There are also some reports on applying DL in the diagnosis of adnexal tumors [25,26,27]. However, other existing models in the literature only distinguished between benign and malignant tumors, and the discrimination of borderline tumors and classification of benign masses into detailed pathological subtypes has not yet been achieved using AI.

Borderline tumors often affect women of reproductive age. An accurate diagnosis is key to selecting the most appropriate surgical approach while preserving fertility [28]. Benign tumors of different pathologies differ in their risk of complications. Thus, detailed classification is crucial in clinical practice [1]. To overcome current diagnostic limitations, we attempted to establish a DL model system that could provide a complete diagnostic interpretation of ultrasound images, including: detecting and locating adnexal masses; distinguishing between benign, borderline, and malignant tumors; and distinguishing the pathological subtypes including endometriomas, other epithelial tumors except endometriomas, germ cell tumors, sex cord-stromal tumors, and inflammation for benign tumors. We also compared the diagnostic performance of the DL model system with that of the expert sonographer SA (recognized as the best method to diagnose adnexal tumors) and intermediate and junior sonographers.

## 2. Materials and Methods

### 2.1. Ethical Approval

In this retrospective study, the use of previously obtained sonographic images was approved by the ethical committees of all participating centers (the first Center of Chinese PLA General Hospital, the 7th Center of Chinese PLA General Hospital, and Hengshui People’s Hospital) via wavier of patient informed consent.

### 2.2. Participants and Datasets

We retrospectively reviewed transvaginal and abdominal ultrasound images taken at the first Center of Chinese PLA General Hospital from 2015 to 2021. The data from 2015 to 2018 were used to create a training dataset while the data from 2019 to 2021 were used as the internal validation dataset. Patients were retrospectively recruited in the 7th Center of Chinese PLA General Hospital from 2020 to 2022 for external test dataset 1, and patients in Hengshui People’s Hospital from 2021 to 2022 were used for external test dataset 2.

Consecutive images from patients with at least one adnexal mass who underwent surgery (providing histological diagnoses for ground truth) and had normal ovaries or ovaries that had been resected previously were eligible for inclusion. Pregnant patients were excluded. Grayscale and color doppler ultrasound images were acquired with Mindray Resona8T, Mindray Resona7, GE VolusonE8, EPIQ7, SAMSUNG, WS80A, HITACHI, and SIEMENS machines equipped with transvaginal and transabdominal probes. The study included only one adnexal mass per patient. If more than one mass was detected, then the mass with the most complex morphology or, in the case of a similar morphology, the largest diameter, was used [16,17].

The pathological results provided the definitive diagnosis. The final pathological diagnosis results were classified into three types: benign, borderline, and malignant tumors. Benign masses were further categorized into five pathological subtypes: endometriomas, other epithelial tumors except endometriomas, germ cell tumors, sex cord-stromal tumors, and inflammation.

### 2.3. Annotation and Framework

A sonographer annotated the adnexal mass and papillary areas on the ultrasound images. The annotation was checked by two expert sonographers and was decided upon their confirmation. The morphological feature of “papillary projection” was defined as any solid projection into the cyst cavity from the cyst wall with a height ≥ 3 mm. The hyper-reflective area in dermoid cysts and “sludge” or blood clots in endometriomas were not regarded as a papillary projection [29]. A cyst with papillary projections is the most significant ultrasound characteristic for borderline tumors [30]. We thus included the additional annotation of papillary projections to discriminate borderline adnexal masses from benign and malignant tumors [26,31,32].

**Figure 1:**
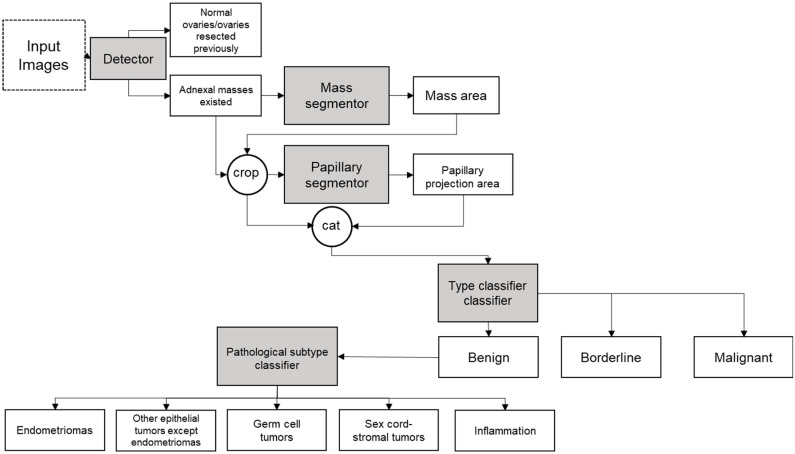
Flowchart of the framework of the DL model system. Images imported into the system are indicated in dotted boxes. Grey boxes show the five parts of the DL model system. Diagnosis results are shown in solid boxes. Images were input into the DL system, and those with adnexal masses were picked out by the detector. The area of masses was recognized by the mass segmentor and papillary projections were located by the papillary segmentor if they existed. The results of these two segmentors and the original images were taken into consideration to make the classification. The type classification would diagnose the adnexal masses into the three types: benign, borderline, and malignant. The borderline and malignant tumors were directly output as the final result. Benign tumors were further distinguished as endometriomas, other epithelial tumors except endometriomas, germ cell tumors, sex cord-stromal tumors, and inflammation.

We designed a deep learning (DL) system containing five models to complete the diagnosis for adnexal masses: a detector, a mass segmentor, a papillary segmentor, a type classifier, and a pathological subtype classifier (Figure 1). First, the detector aimed to find the ovaries with adnexal masses while discarding normal ovaries or ovaries resected. The detector focused on two-dimensional grayscale images because color doppler flow images are not available in normal ovaries. Second, the mass segmentor located the area of the tumor. Third, for masses with papillary projections, the papillary segmentor located the area of papillary projections based on the mass area. Fourth, the type classifier predicted the type (benign, borderline, or malignant) of the adnexal masses based on the information for the mass area, papillary area, and original images. Finally, if the type was benign, the pathological subtype classifier inferred the detailed pathology of the adnexal masses. The borderline and malignant types were output directly as the final result. No human intervention was performed in the entire process. All DL models except the detector were compatible with both two-dimensional grayscale images and color doppler flow images; the detector used only two-dimensional grayscale images.

### 2.4. Model Architecture

The backbone structure of the detector was LKResnet-18, which is a combination of ResNet-18 [33] and LKNet [34]. Resnet-18 consists of eight residual blocks, each of which contains two convolutional layers and a residual link. Each convolution layer of ResNet-18 has a convolution kernel size of 3 × 3 (excluding the first convolutional layer), and the small convolution kernel can only focus on the texture features of the image. In contrast, the larger convolution kernel can focus on the shape information of the image, while the human recognition of objects is based more on shape cues than texture cues [34]. We used the convolution kernel size of 15 × 15 instead of 3 × 3. At the same time, we use depthwise convolution to balance the kernel size and GPU memory. In addition, we replaced the ReLU activation function and batch normalization with the more efficient and robust GeLU and layer normalization.

We adopted LKResnet-18 for two reasons: first, a small convolutional kernel might lead to extracting texture-based features rather than structure-based features while large convolutional kernels, such as 15 × 15 and 31 × 31 kernels, can achieve better performance [32]; second, for ultrasound images, the texture of the image can provide limited information, and the structure and contour information is of much importance. Therefore, the large kernel convolutional network (LKNet) was a very suitable solution.

The convolutional layers could extract 512 latent features from each image; then a fully-connected classifier identified the probability of having a tumor in the image based on the pattern of latent features. In the training stage, we adopted the following data augmentations to improve the robustness of the model: random shift, random scale, and random rotation. The model parameters were updated in 20,000 iterations with a batch size of eight. The loss function adopted cross-entropy, which is defined as:

(1) $$L_{\mathrm{ce}}=-\frac{1}{N}\overset{N}{\underset{i}{\sum }}\left[y^{\left(i\right)}\log\left(p(x^{\left(i\right)}|\theta )\right)\right],$$

where *N* is the number of samples, θ is the parameter of the detector model, *y*^(i)^ represents the tumor label of sample I, and *p*(x^(i)^|θ) is the probability of the tumor prediction x^(i)^ of sample i.

The purpose of the mass segmentor was to segment the specific location of the tumor in the image. The segmentor model structure was U-net [35] and the backbone was LKResnet-18. As shown in the Table S1, we compared the performances of different segmentation models. It turned out that U-net with LKResnet-18 outperformed the other segmentation models. U-net contains a decoder and an encoder; the former extracted high-level semantic features of the image and reduced the resolution while the latter fused high-level semantic features with low-level texture features and restored the resolution. In the training stage, we adopted the following data augmentations: random shift, random scale, random rotate, horizontal flip, and vertical flip. The model parameters were updated in 20,000 iterations with a batch size of four. The loss function adopted cross-entropy loss and boundary loss [36]. Boundary loss used boundary matching to supervise the segmentor. The settings for the mass segmentor were also used for the papillary segmentor.

**Figure 2:**
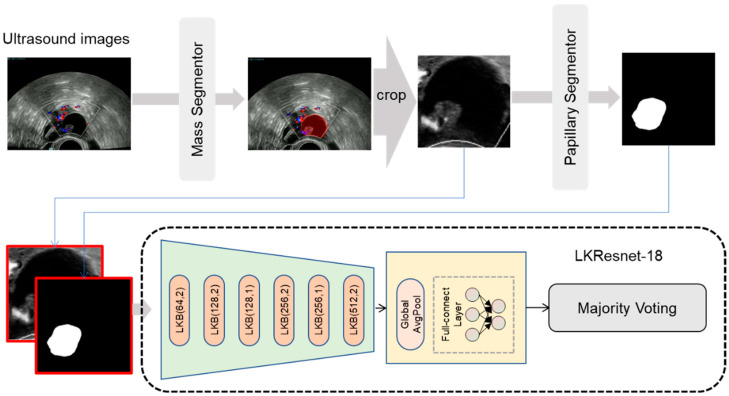
The framework of the type and pathological subtype classifiers. “LKB (c,s)” refers to a basic large kernel block containing two depthwise–pointwise convolutions with output channels of c and strides of s. For each image, including color doppler flow images and two-dimensional sonographic images, the mass segmentor was used to locate the tumor region. Then the image was cropped to just the tumor region. The papillary segmentor was used to locate the papillary region in the tumor area. The papillary region and tumor region were concatenated as the input into the classifier.

The model structure of the type classifier and pathological subtype classifier was LKResnet-18, as shown in Figure 2. The input to the classifier was the combination of the outputs of the mass segmentor and papillary segmentor. We adopted the following data augmentations for training: random shift, random scale, random rotation, Gaussian blur, random brightness, random contrast, horizontal flip, and vertical flip. The model parameters were updated in 10,000 iterations with a batch size of four. The loss function adopted cross-entropy loss as given in Equation (1).

Each case contained multiple two-dimensional sonographic images and color doppler flow images. The type and pathological subtype classifiers scored each image, which provided multiple predictions for each case. As shown in Figure 2, the model used majority voting, a rule-based case-wise strategy, to generate the case-wise prediction.

The five models used the AdamW [37,38] optimizer with a learning rate of 3 × 10^−5^ and weight L2 regularization of 3 × 10^−4^. All experiments were performed on two NVIDIA GeForce RTX 3090 graphics processing units.

### 2.5. Evaluation and Comparison with Sonographers

The diagnostic performance of the DL system containing five models was evaluated using the internal validation dataset, external test dataset 1, and external test dataset 2. Three reviewers assessed all images in the external test set 1 and external set 2 independently. All reviewers were certificated sonographers. Reviewer A was an expert gynecological sonographer with 37 years of clinical experience. Reviewer B was an intermediate sonographer with 16 years of experience. Reviewer C had 2 years of clinical experience as a junior sonographer. All reviewers were blinded to the clinical information, original ultrasound reports, and pathological results. The diagnostic performance of the sonographers was evaluated and compared with the DL model system in external test sets.

### 2.6. Statistical Analysis

Continuous measures are presented as mean. Categorical measures are presented as proportion with 95% CIs and were compared using the chi-squared test. The frequency of the morphological characteristic of papillary projection for different adnexal mass types was compared using pairwise differences.

We evaluated the diagnostic efficiency of the DL system based on the discrimination and calibration performance. For the detector, accuracy in identifying adnexal masses was measured. The dice score was computed to estimate the performance of the mass and papillary segmentors. We constructed the confusion matrix for the type classifier and the pathological subtype classifier. The accuracy, sensitivity, specificity, positive predictive value, negative predictive value, and macro-F1 scores were calculated for both classifiers [39]. The calibration plot and the Brier score were used to estimate the calibration of the type classifier and the pathological subtype classifier.

The accuracy, sensitivity, specificity, positive predictive value, negative predictive value, and macro-F1 scores of the sonographers were also computed for comparison with the DL system. Statistical analysis was done using Python; *p* < 0.05 was considered to indicate a statistically significant difference.

## 3. Results

### 3.1. Data and Patients

A total of 1099 cases (4497 images) were included in the training dataset and 460 cases (1217 images) in the internal validation set. External test set 1 contained 490 images of 198 cases; external test set 2 had 761 images of 264 cases. The baseline characteristics of each dataset are shown in Table 1.

### 3.2. Papillary Projections

Papillary projections are more frequent in borderline than in benign and malignant tumors. Benign and malignant tumors do not differ in papillary projection frequency.

In the training dataset, the frequency of papillary projection was 5.61% (21/374) in benign adnexal masses, 54.00% (27/50) in borderline tumors, and 8.98% (15/167) in malignant tumors. Borderline tumors had a higher rate of papillary projections than benign tumors (*p* < 0.001) and malignant tumors (*p* < 0.001). There was no statistical difference in the presence of papillary projections between benign and malignant tumors (*p* = 0.147).

In the internal validation dataset, papillary projections presented in 5.22% (7/134) of benign tumors, 66.67% (10/15) of borderline tumors, and 12.50% (7/56) of malignant tumors. The frequency was higher in borderline tumors than in benign (*p* < 0.001) and malignant tumors (*p* < 0.001). There was no statistical difference between benign and malignant tumors (*p* = 0.080).

In external test dataset 1, 5.88% (3/51) of benign tumors and 50% (4/8) of borderline tumors showed the morphological characteristic of papillary projection, while no malignant tumors presented with that ultrasound feature. There was a higher rate of papillary projections in borderline tumors than benign (*p* < 0.001) or malignant tumors (*p* < 0.001). No statistical difference was detected between benign and malignant tumors (*p* = 0.106).

In external test dataset 2, the rate of papillary projections was 3.17% (4/126) in benign tumors, 75.00% (6/8) in borderline tumors, and 12.00% (3/25) malignant tumors. More borderline tumors had papillary projections than benign (*p* < 0.001) and malignant tumors (*p* < 0.001). There was no statistical difference between benign and malignant tumors (*p* = 0.055).

### 3.3. Diagnostic Performance for the DL Model System

The performance of each of the five models in the DL system was considered in turn. The detector was implemented in the training test to distinguish sonographic images with adnexal masses from those without lesions. The detector performed very well, achieving an accuracy of 0.965 (95% CI: 0.933–0.975) on the internal validation dataset. For external test dataset 1, the accuracy was 0.943 (0.922–0.951); for external dataset 2, the accuracy was 0.931 (0.919–0.952).

The mass segmentor was created to ascertain the location of the adnexal masses. It performed well, achieving a dice score of 0.945 in the internal validation dataset. It remained stable in the multicenter test datasets. The dice score for the mass segmentor was 0.923 for external test dataset 1 and 0.912 for external test dataset 2.

**Figure 3:**
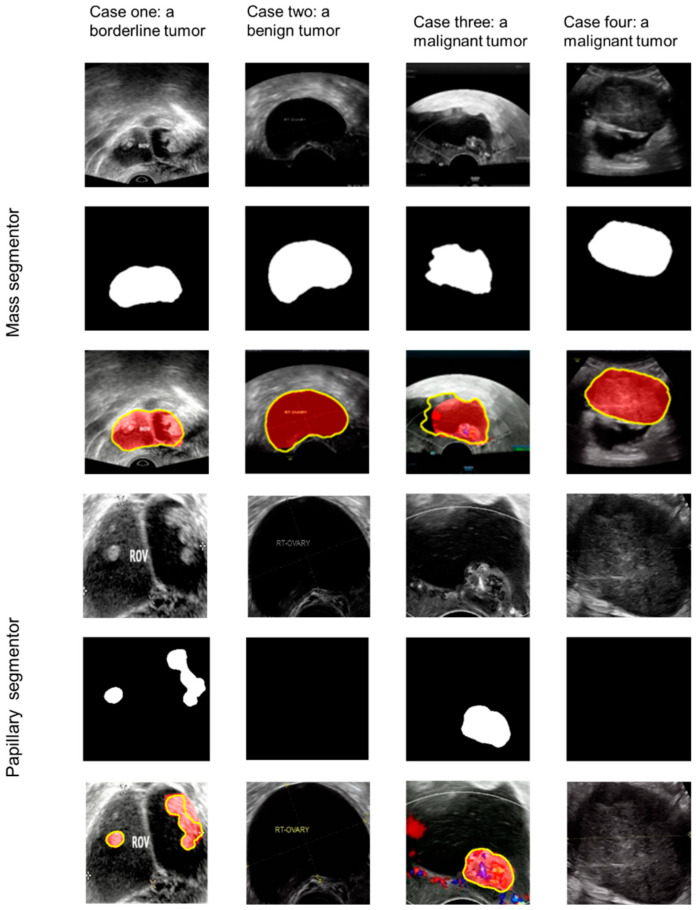
Case one shows a borderline tumor with papillary projections; case two shows a benign tumor without papillary projections; case three shows a malignant tumor with a papillary projection; case four shows a malignant tumor without papillary projections. The top three rows show the process of the mass segmentor; the three rows below show the process of the papillary segmentor. Yellow line: the result of the mass segmentor or the papillary segmentor; red area: ground truth.

The papillary projection segmentor located the projections accurately. The dice score was 0.864 for the internal validation dataset, 0.852 for external test dataset 1, and 0.855 for external test dataset 2. Figure 3 shows the results of four cases of the mass segmentor and the papillary segmentor.

**Figure 4:**
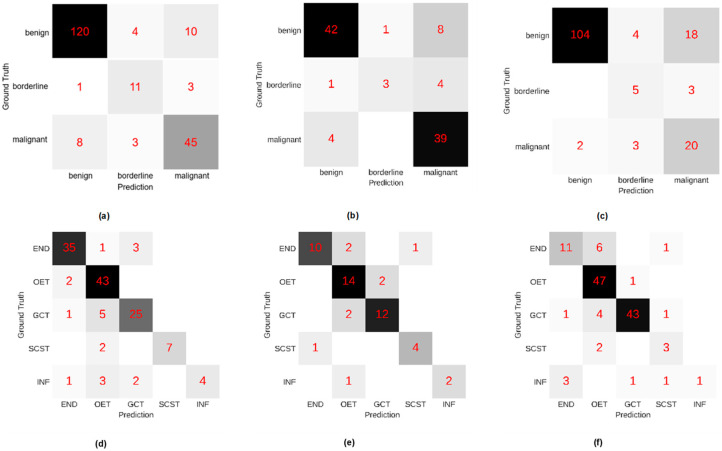
Confusion matrixes of the type classifier for (**a**) the internal validation dataset, (**b**) external test dataset 1, and (**c**) external test dataset 2. Confusion matrixes of the pathological subtype classifier for (**d**) the internal validation dataset, (**e**) external test dataset 1, and (**f**) external test dataset 2. The lateral axis represents the predictive result of the type classifier or the pathological subtype classifier; the vertical axis represents the ground truth (pathological diagnosis) of the adnexal masses. END: endometriosis; OET: other epithelial tumors except endometriosis; GCT: germ cell tumors; SCST: sex cord-stromal tumors; INF: inflammation.

The type classifier was used to classify adnexal masses as benign, borderline, and malignant. According to the confusion matrixes of the type classifier in Figure 4, most predicted results of the adnexal masses were in accordance with the ground truth. For the internal validation dataset, the macro-F1 score was 0.791. For external test dataset 1, the classifier had a macro-F1 score of 0.749. For external test dataset 2, the macro-F1 score was 0.684.

The pathological subtype classifier could discriminate among the five pathological subtypes of benign adnexal tumors. The results were visualized in the confusion matrix shown in Figure 4. The macro-F1 score in the internal validation dataset to distinguish among endometriomas, other epithelial tumors except endometriomas, germ cell tumors, sex cord-stromal tumors, and inflammation was 0.831. For external dataset 1, the macro-F1 score was 0.826. For external dataset 2, the macro-F1 score of the pathological subtype classifier was 0.714.

**Figure 5:**
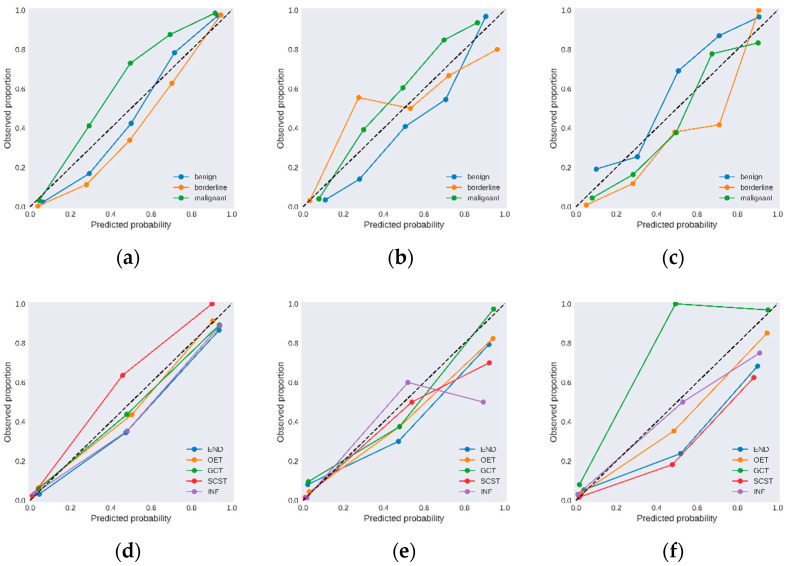
Calibration plots of the type classifier for (**a**) the internal validation dataset, (**b**) external test dataset 1, and (**c**) external test dataset 2. Calibration plots of the pathological subtype classifier for (**d**) the internal validation dataset, (**e**) external test dataset 1, and (**f**) external test dataset 2.

**Figure 6:**
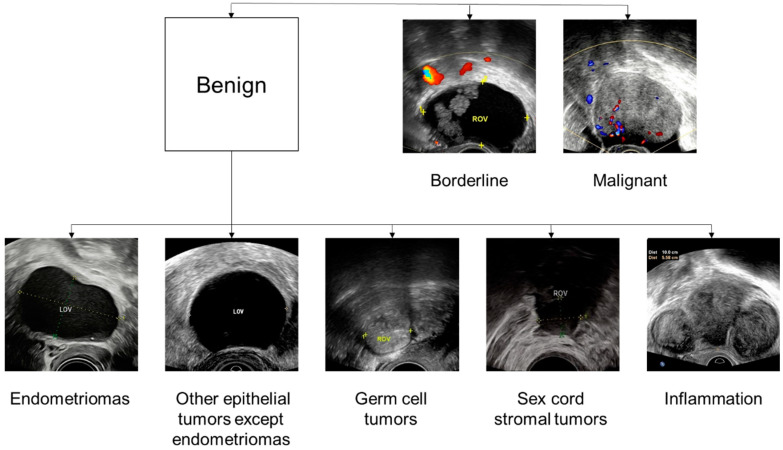
Example ultrasound images of different types of adnexal masses and different pathological subtypes of benign tumors.

Comprehensive diagnostic performance parameters of the type classifier and the pathological subtype classifier are presented in Table 2 and Table 3. The calibration plots further indicated that the type classifier and the pathological subtype classifier fit the data very well (Figure 5). The Brier score of the type classifier was 0.090 for the internal validation dataset, 0.102 for external test dataset 1, and 0.082 for external test dataset 2. The Brier score for the pathological subtype classifier was 0.056 for the internal validation dataset, 0.064 for external test dataset 1, and 0.058 for external test dataset 2. Figure 6 shows the final classification of the type classifier and the pathological subtype classifier.

### 3.4. Comparison with Sonographers

The diagnostic performance parameters of three reviewers with different experience were evaluated using the external test datasets (Table 4).

For discrimination of benign tumors, the DL system had a higher accuracy (0.863 vs. 0.578, *p* < 0.001), sensitivity (0.824 vs. 0.451, *p* < 0.001), and specificity (0.902 vs. 0.706, *p* = 0.013) than the junior reviewer for external test dataset 1. For external test dataset 2, the specificity of the DL system for benign tumors was higher than that of the expert (0.939 vs. 0.606, *p* = 0.001) and the junior sonographer (0.939 vs. 0.697, *p* = 0.011) but the sensitivity was lower than that of the expert sonographer (0.825 vs. 0.921, *p* = 0.023).

For classification of borderline tumors, the DL system achieved a higher sensitivity (0.625 vs. 0.000, *p* = 0.026) than that of the junior reviewer in external test dataset 2.

For classifying malignant tumors, the accuracy, sensitivity, and specificity of the DL system also exceeded that of the junior reviewer (accuracy: 0.843 vs. 0.559, *p* < 0.001; sensitivity: 0.907 vs. 0.698, *p* = 0.015; specificity: 0.797 vs. 0.458, *p* < 0.001) in external test dataset 1. The specificity of the DL system was lower than that of the expert sonographer (0.797 vs. 0.932, *p* = 0.031) when discriminating malignant tumors in external dataset 1. The specificity of the DL system was lower than that of the expert reviewer when classifying malignant tumors in external test dataset 2 (0.843 vs. 0.940, *p* = 0.011).

In the external test datasets, the diagnostic performance of the DL system was comparable to that of the intermediate sonographer.

## 4. Discussion

We established a DL system that comprised five models (a detector, a mass segmentor, a papillary segmentor, a type classifier, and a pathological subtype classifier) to automatically diagnose adnexal masses in sonographic images. The DL model system could identify the existence of the tumor and recognize the area of the mass and papillary projection precisely. Masses were correctly classified into benign, borderline, and malignant tumors; benign tumors were further categorized into one of five pathological subtypes. Our DL system had the ability to complete multiple tasks. This allowed the DL model system to automatically implement the complete diagnostic process for adnexal masses and provide abundant information for clinical therapy [31].

The DL system showed good discrimination in the internal validation dataset and the external test datasets 1 and 2 with macro-F1 scores of 0.791, 0.749, and 0.684, respectively. According to the confusion matrixes, the DL system was able to distinguish benign and malignant adnexal tumors precisely. The discrimination between benign and malignant tumors could also be achieved through deep learning in other studies [22]. However, previous DL models for adnexal masses never discriminated borderline tumors from benign and malignant mimics. The ability to discriminate borderline tumors is essential in choosing the appropriate treatment for patients with adnexal masses. Fertility-sparing surgery is the gold standard therapeutic modality for women with borderline tumors who wish to maintain their fertility without impacting overall survival [28,40]. If borderline tumors are misdiagnosed as benign tumors, clinical treatment may be delayed [30]. The commonly used radical surgery for patients with malignant tumors may be excessive given that borderline tumors have a better overall 5-year survival than malignant tumors [4,28]. Therefore, we tried to discern borderline tumors from benign and malignant tumors by annotating morphological features of the masses, which could direct the DL models to extract more valuable imaging information for medical diagnosis. The papillary projection was a morphological characteristic that appeared more frequently in borderline tumors than in benign or malignant tumors (*p* < 0.001). In addition, papillary projections could be segmented successfully in our study. Accurate information on papillary projections might allow the DL system to improve its ability to distinguish borderline tumors. In this study, we correctly classified 11 of 15 borderline tumors in the internal validation dataset. However, the type classifier was not reliable in the external test datasets. According to the confusion matrixes, there were only three of eight and five of eight borderline tumors diagnosed correctly in the two respective external datasets. Moreover, most of the borderline tumors that were misdiagnosed were predicted as malignant tumors. In previous studies in which borderline adnexal tumors were difficult to discriminate, it was recognized that borderline tumors should be classified as malignant tumors to improve the survival rates. In this condition, the poor performance in classifying borderline tumors may be acceptable. We reviewed all the borderline tumors that were misdiagnosed in the external datasets. All nine cases diagnosed as malignant tumors presented as multilocular cysts with or without a solid component. Therefore, further study regarding adnexal masses with multiple septations may improve the distinguishment between borderline and malignant tumors.

Observation is preferred for patients with benign adnexal tumors to minimize the potential risks and complications of surgery [2]. During observation, 20.2–39% of benign tumors spontaneously resolve and the risk of malignancy is lower than 0.5% [1,41,42]. However, the risk of potential complications such as cyst rupture and torsion and the presence of clinical symptoms remain the primary reasons for surgery [1]. Different subtypes of benign adnexal masses have various complications, and doctors should pay attention to the corresponding complications of each subtype of benign tumors. Thus, it is significant in clinical work to distinguish among different pathological subtypes of benign tumors. Morphological features of benign tumors were previously used only for excluding malignant tumors, but they can also help with classification into detailed pathological subtypes. The subtype classifier of the DL system was able to discern most subtypes of benign tumors with macro F1-scores of 0.831 in the internal validation dataset, 0.826 in external test dataset 1, and 0.714 in external test dataset 2. The pathological subtype classifier performed well when distinguishing endometriomas, other epithelial tumors except endometriomas, germ cell tumors, and sex cord-stromal tumors: the diagnostic parameters were satisfactory, as shown in Table 3. An accurate diagnosis may assist with the decision on whether an operation is necessary. When endometriomas are diagnosed, intervention is advised for those with unrelieved pelvic pain and desire for fertility, and follow-up is required to survey for recurrence [7]. Dermoid cysts (benign germ cell tumors) are the most frequent adnexal mass to twist. This accurate discrimination can prevent potential ovarian torsion via a timely operation, especially for those tumors with larger diameter. In contrast, torsion does not often occur in endometriomas or malignant tumors [8]. The precise diagnosis of sex cord-stromal tumors may allow clinical signs of hormonal production such as virilization, precocious puberty, and menstrual changes to be explained and settled [9]. When inflammation is diagnosed, the proper anti-inflammatory drug therapy can effectively relieve symptoms, preventing unnecessary surgery. However, the ultrasound manifestations of inflammation present variously at different stages of the disease. In addition, the low rate of inflammation among benign tumors also increases the difficulty in discriminating inflammation reliably. The sensitivity was only 0.400 in the internal validation dataset and 0.167 in the external dataset 2. In real clinical work, experienced sonographers would take the patients’ clinical symptoms into consideration to assist in the diagnosis of inflammation.

The DL system had an excellent diagnostic performance, exceeding the accuracy and sensitivity of the junior sonographer and matching that of the intermediate and expert sonographers. The sensitivity of the DL system to benign and malignant tumors was higher than that of the junior sonographer in external test dataset 1 and the sensitivity to borderline tumors was higher for the DL system in external test dataset 2. Using the DL system would thus be practical in clinical work and improve the ability of a sonographer lacking experience. Moreover, the stability of the DL system was proved by testing it using external datasets from different centers.

Chen et.al established DL algorithms to distinguish malignant from benign adnexal tumors with a diagnostic performance comparable to expert subjective assessment, similar to our study [26]. However, Chen’s study did not involve the distinguishment of borderline tumors or a comparison with expert assessments.

Our DL system had some limitations. First, we hoped to separate primary adnexal cancer from secondary metastasis cancer, but our DL model was unsuccessful at achieving this. The insufficient number of cases of secondary metastasis cancer was likely the key reason for this. In addition, more morphological features had to be found to distinguish primary from metastatic adnexal cancer. Second, the performance of the DL system in discerning borderline tumors and inflammation were not satisfactory enough. More valuable ultrasound characteristics for borderline tumors and a larger sample may improve this condition. Third, this was a retrospective study, so prospective external validation needs to be implemented in the future.

## 5. Conclusions

In this multicenter study, we implemented a DL model system to perform the complete diagnostic process for adnexal masses. The masses were detected and segmented automatically and classified into benign, borderline, and malignant types. The benign tumors were then further classified into different pathological subtypes. This DL system matched the abilities of expert and intermediate sonographers and outperformed the junior sonographer.

## Figures and Tables

**Table 1:** Baseline characteristics of datasets in the study.

Patients	Type Classification	Pathological Subtype Classification	Training Dataset	Internal Validation Dataset	External Test Dataset 1	External Test Dataset 2
Mean age			43.43 (11, 81)	44.50 (11, 81)	48.87 (15, 77)	43.26 (19, 50)
Healthy cases * (images)			508 (1100)	255 (564)	96 (178)	105 (233)
Cases (images) with adnexal masses ^†^			591 (3397)	205 (653)	102 (312)	159 (528)
	Benign		374 (1117)	134 (377)	51 (151)	126 (401)
		END ^†^	117 (332)	39 (102)	13 (35)	18 (51)
		OET	103 (342)	45 (127)	16 (49)	48 (157)
		GCT	102 (272)	31 (82)	14 (50)	49 (156)
		SCST	33 (107)	9 (32)	5 (11)	5 (18)
		INF	19 (64)	10 (34)	3 (6)	6 (19)
	Borderline		50 (917)	15 (57)	8 (27)	8 (30)
	Malignant		167 (1363)	56 (219)	43 (134)	25 (97)
Total			1099 (4497)	460 (1217)	198 (490)	264 (761)

The age is given as the mean value (min, max); the number of cases (number of images) is given for each category. * Healthy cases: cases with normal ovaries and ovaries resected previously. ^†^ END: endometrioma; OET: other epithelial tumors except endometrioma; GCT: germ cell tumors; SCST: sex cord-stromal tumors; INF: inflammation.

**Table 2:** Diagnostic performance parameters for the type classifier.

Variable	Internal Validation Dataset			External Test Dataset 1			External Test Dataset 2		
	Benign	Borderline	Malignant	Benign	Borderline	Malignant	Benign	Borderline	Malignant
Accuracy	0.888 (0.875–0.902)	0.946 (0.940–0.957)	0.883 (0.875–0.897)	0.863 (0.846–0.879)	0.941 (0.934–0.956)	0.843 (0.824–0.868)	0.849 (0.832–0.867)	0.937 (0.930–0.951)	0.836 (0.825–0.853)
Sensitivity	0.896 (0.882–0.913)	0.733 (0.667–0.786)	0.804 (0.780–0.837)	0.824 (0.795–0.864)	0.375 (0.286–0.500)	0.907 (0.892–0.944)	0.825 (0.809–0.847)	0.625 (0.571–0.714)	0.800 (0.762–0.857)
Specificity	0.873 (0.855–0.902)	0.963 (0.959–0.971)	0.913 (0.902–0.926)	0.902 (0.886–0.933)	0.989 (0.988–1.000)	0.797 (0.769–0.830)	0.939 (0.926–0.967)	0.954 (0.948–0.964)	0.843 (0.828–0.860)
Positive predictive value	0.930 (0.921–0.947)	0.611 (0.563–0.688)	0.776 (0.750–0.811)	0.894 (0.875–0.927)	0.750 (0.667–1.000)	0.765 (0.733–0.800)	0.981 (0.978–0.990)	0.417 (0.364–0.500)	0.488 (0.444–0.528)
Negative predictive value	0.816 (0.794–0.843)	0.979 (0.976–0.983)	0.925 (0.916–0.939)	0.836 (0.813–0.872)	0.949 (0.943–0.966)	0.922 (0.909–0.953)	0.584 (0.551–0.625)	0.980 (0.977–0.985)	0.958 (0.952–0.972)

**Table 3:** Diagnostic performance parameters for the pathological subtype classifier.

Variable	Internal Validation Dataset					External Test Dataset 1					External Test Dataset 2				
	END	OET	GCT	SCST	INF	END	OET	GCT	SCST	INF	END	OET	GCT	SCST	INF
Accuracy	0.940 (0.933–0.950)	0.903 (0.892–0.917)	0.918 (0.908–0.933)	0.985 (0.983–0.992)	0.955 (0.950–0.967)	0.922 (0.911–0.956)	0.863 (0.844–0.889)	0.922 (0.911–0.956)	0.961 (0.956–0.978)	0.980 (0.978–1.000)	0.913 (0.903–0.929)	0.897 (0.885–0.912)	0.937 (0.929–0.947)	0.960 (0.956–0.973)	0.960 (0.956–0.973)
Sensitivity	0.897 (0.879–0.939)	0.956 (0.947–0.976)	0.806 (0.778–0.852)	0.778 (0.714–0.875)	0.400 (0.333–0.500)	0.769 (0.700–0.833)	0.875 (0.846–0.933)	0.857 (0.818–0.923)	0.800 (0.667–1.000)	0.667 (0.500–1.000)	0.611 (0.538–0.688)	0.979 (0.976–1.000)	0.878 (0.857–0.907)	0.600 (0.500–0.750)	0.167 (0.000–0.250)
Specificity	0.958 (0.952–0.976)	0.876 (0.859–0.899)	0.951 (0.944–0.967)	1.000 (1.000–1.000)	1.000 (1.000–1.000)	0.974 (0.969–1.000)	0.857 (0.833–0.900)	0.946 (0.935–0.970)	0.978 (0.975–1.000)	1.000 (1.000–1.000)	0.962 (0.958–0.979)	0.846 (0.826–0.871)	0.974 (0.970–0.986)	0.975 (0.972–0.982)	1.000 (1.000–1.000)
Positive predictive value	0.897 (0.879–0.938)	0.796 (0.771–0.833)	0.833 (0.808–0.885)	1.000 (1.000–1.000)	1.000 (1.000–1.000)	0.909 (0.875–1.000)	0.737 (0.688–0.813)	0.857 (0.818–0.923)	0.800 (0.667–1.000)	1.000 (1.000–1.000)	0.733 (0.667–0.800)	0.797 (0.769–0.830)	0.956 (0.947–0.976)	0.500 (0.400–0.600)	1.000 (1.000–1.000)
Negative predictive value	0.958 (0.952–0.976)	0.975 (0.971–0.986)	0.942 (0.934–0.957)	0.984 (0.982–0.991)	0.954 (0.948–0.966)	0.925 (0.912–0.946)	0.938 (0.926–0.966)	0.946 (0.935–0.970)	0.978 (0.975–1.000)	0.980 (0.977–1.000)	0.937 (0.929–0.950)	0.985 (0.982–1.000)	0.926 (0.915–0945)	0.983 (0.981–0.991)	0.960 (0.955–0.973)

END: endometriosis; OET: other epithelial tumors except endometriosis; GCT: germ cell tumors; SCST: sex cord-stromal tumors; INF: inflammation.

**Table 4:** Diagnostic performance of sonographers using external test datasets.

Variables	Benign						Borderline						Malignant					
	External Test Dataset 1			External Test Dataset 2			External Test Dataset 1			External Test Dataset 2			External Test Dataset 1			External Test Dataset 2		
	Reviewer A	Reviewer B	Reviewer C	Reviewer A	Reviewer B	Reviewer C	Reviewer A	Reviewer B	Reviewer C	Reviewer A	Reviewer B	Reviewer C	Reviewer A	Reviewer B	Reviewer C	Reviewer A	Reviewer B	Reviewer C
Accuracy	0.814	0.843	0.578	0.855	0.818	0.780	0.892	0.912	0.902	0.943	0.893	0.918	0.882	0.873	0.559	0.899	0.824	0.811
Sensitivity	0.824	0.784	0.451	0.921	0.825	0.802	0.500	0.375	0.000	0.250	0.125	0.000	0.814	0.930	0.698	0.680	0.680	0.760
Specificity	0.804	0.902	0.706	0.606	0.788	0.697	0.926	0.957	0.978	0.980	0.934	0.967	0.932	0.831	0.458	0.940	0.851	0.821
Positive predictive value	0.808	0.889	0.605	0.899	0.937	0.910	0.364	0.429	0.000	0.400	0.091	0.000	0.897	0.800	0.484	0.680	0.459	0.442
Negative predictive value	0.820	0.807	0.563	0.667	0.542	0.479	0.956	0.947	0.920	0.961	0.953	0.948	0.873	0.942	0.675	0.940	0.934	0.948

## Data Availability

The data presented in this study are available on request from the corresponding authors (Q.L. and L.W.). The data are not publicly available due to hospital regulations.

## Supplementary Materials

The following supporting information can be downloaded at: https://www.mdpi.com/article/10.3390/cancers14215291/s1, Table S1: The comparison of dices among different segmentation models. References [43,44] are cited in the Supplementary Materials.

## References

[1] Froyman W., Landolfo C., De Cock B., Wynants L., Sladkevicius P., Testa A., Van Holsbeke C., Domali E., Fruscio R., Epstein E. (2019). Risk of complications in patients with conservatively managed ovarian tumours (IOTA5): A 2-year interim analysis of a multicentre, prospective, cohort study. Lancet. Oncol..

[2] American College of Obstetricians and Gynecologists’ Committee on Practice Bulletins—Gynecology (2016). Practice Bulletin No. 174: Evaluation and Management of Adnexal Masses. Obstet. Gynecol..

[3] Andreotti R., Timmerman D., Strachowski L., Froyman W., Benacerraf B., Bennett G., Bourne T., Brown D., Coleman B., Frates M. (2020). O-RADS US Risk Stratification and Management System: A Consensus Guideline from the ACR Ovarian-Adnexal Reporting and Data System Committee. Radiology.

[4] Heintz A., Odicino F., Maisonneuve P., Quinn M., Benedet J., Creasman W., Ngan H., Pecorelli S., Beller U. (2006). Carcinoma of the ovary. FIGO 26th Annual Report on the Results of Treatment in Gynecological Cancer. Int. J. Gynaecol. Obstet. Off. Organ Int. Fed. Gynaecol. Obstet..

[5] Armstrong D., Alvarez R., Bakkum-Gamez J., Barroilhet L., Behbakht K., Berchuck A., Chen L., Cristea M., DeRosa M., Eisenhauer E. (2021). Ovarian Cancer, Version 2.2020, NCCN Clinical Practice Guidelines in Oncology. J. Natl. Compr. Cancer Netw. JNCCN.

[6] Timmerman D., Testa A., Bourne T., Ameye L., Jurkovic D., Van Holsbeke C., Paladini D., Van Calster B., Vergote I., Van Huffel S. (2008). Simple ultrasound-based rules for the diagnosis of ovarian cancer. Ultrasound Obstet. Gynecol. Off. J. Int. Soc. Ultrasound Obstet. Gynecol..

[7] Chapron C., Marcellin L., Borghese B., Santulli P. (2019). Rethinking mechanisms, diagnosis and management of endometriosis. Nat. Rev. Endocrinol..

[8] Anfelter P., Testa A., Chiappa V., Froyman W., Fruscio R., Guerriero S., Alcazar J., Mascillini F., Pascual M., Sibal M. (2020). Imaging in gynecological disease (17): Ultrasound features of malignant ovarian yolk sac tumors (endodermal sinus tumors). Ultrasound Obstet. Gynecol. Off. J. Int. Soc. Ultrasound Obstet. Gynecol..

[9] Schultz K., Harris A., Schneider D., Young R., Brown J., Gershenson D., Dehner L., Hill D., Messinger Y., Frazier A. (2016). Ovarian Sex Cord-Stromal Tumors. J. Oncol. Pract..

[10] Bristow R., Smith A., Zhang Z., Chan D., Crutcher G., Fung E., Munroe D. (2013). Ovarian malignancy risk stratification of the adnexal mass using a multivariate index assay. Gynecol. Oncol..

[11] Moore R., Brown A., Miller M., Skates S., Allard W., Verch T., Steinhoff M., Messerlian G., DiSilvestro P., Granai C. (2008). The use of multiple novel tumor biomarkers for the detection of ovarian carcinoma in patients with a pelvic mass. Gynecol. Oncol..

[12] Prat J. (2014). Staging classification for cancer of the ovary, fallopian tube, and peritoneum. Int. J. Gynaecol. Obstet. Off. Organ Int. Fed. Gynaecol. Obstet..

[13] Alcázar J., Pascual M., Graupera B., Aubá M., Errasti T., Olartecoechea B., Ruiz-Zambrana A., Hereter L., Ajossa S., Guerriero S. (2016). External validation of IOTA simple descriptors and simple rules for classifying adnexal masses. Ultrasound Obstet. Gynecol. Off. J. Int. Soc. Ultrasound Obstet. Gynecol..

[14] Amor F., Vaccaro H., Alcázar J., León M., Craig J., Martinez J. (2009). Gynecologic imaging reporting and data system: A new proposal for classifying adnexal masses on the basis of sonographic findings. J. Ultrasound Med. Off. J. Am. Inst. Ultrasound Med..

[15] Basha M., Refaat R., Ibrahim S., Madkour N., Awad A., Mohamed E., El Sammak A., Zaitoun M., Dawoud H., Khamis M. (2019). Gynecology Imaging Reporting and Data System (GI-RADS): Diagnostic performance and inter-reviewer agreement. Eur. Radiol..

[16] Timmerman D., Testa A., Bourne T., Ferrazzi E., Ameye L., Konstantinovic M., Van Calster B., Collins W., Vergote I., Van Huffel S. (2005). Logistic regression model to distinguish between the benign and malignant adnexal mass before surgery: A multicenter study by the International Ovarian Tumor Analysis Group. J. Clin. Oncol. Off. J. Am. Soc. Clin. Oncol..

[17] Van Calster B., Van Hoorde K., Valentin L., Testa A., Fischerova D., Van Holsbeke C., Savelli L., Franchi D., Epstein E., Kaijser J. (2014). Evaluating the risk of ovarian cancer before surgery using the ADNEX model to differentiate between benign, borderline, early and advanced stage invasive, and secondary metastatic tumours: Prospective multicentre diagnostic study. BMJ Clin. Res. Ed..

[18] Kaijser J., Sayasneh A., Van Hoorde K., Ghaem-Maghami S., Bourne T., Timmerman D., Van Calster B. (2014). Presurgical diagnosis of adnexal tumours using mathematical models and scoring systems: A systematic review and meta-analysis. Hum. Reprod. Update.

[19] Sayasneh A., Ferrara L., De Cock B., Saso S., Al-Memar M., Johnson S., Kaijser J., Carvalho J., Husicka R., Smith A. (2016). Evaluating the risk of ovarian cancer before surgery using the ADNEX model: A multicentre external validation study. Br. J. Cancer.

[20] Van Calster B., Valentin L., Froyman W., Landolfo C., Ceusters J., Testa A., Wynants L., Sladkevicius P., Van Holsbeke C., Domali E. (2020). Validation of models to diagnose ovarian cancer in patients managed surgically or conservatively: Multicentre cohort study. BMJ Clin. Res. Ed..

[21] Arnaout R., Curran L., Zhao Y., Levine J., Chinn E., Moon-Grady A. (2021). An ensemble of neural networks provides expert-level prenatal detection of complex congenital heart disease. Nat. Med..

[22] Gulshan V., Peng L., Coram M., Stumpe M., Wu D., Narayanaswamy A., Venugopalan S., Widner K., Madams T., Cuadros J. (2016). Development and Validation of a Deep Learning Algorithm for Detection of Diabetic Retinopathy in Retinal Fundus Photographs. JAMA.

[23] Cho S., Sun S., Mun J., Kim C., Kim S., Cho S., Youn S., Kim H., Chung J. (2020). Dermatologist-level classification of malignant lip diseases using a deep convolutional neural network. Br. J. Dermatol..

[24] Li X., Zhang S., Zhang Q., Wei X., Pan Y., Zhao J., Xin X., Qin C., Wang X., Li J. (2019). Diagnosis of thyroid cancer using deep convolutional neural network models applied to sonographic images: A retrospective, multicohort, diagnostic study. Lancet. Oncol..

[25] Gao Y., Zeng S., Xu X., Li H., Yao S., Song K., Li X., Chen L., Tang J., Xing H. (2022). Deep learning-enabled pelvic ultrasound images for accurate diagnosis of ovarian cancer in China: A retrospective, multicentre, diagnostic study. Lancet Digit. Health.

[26] Chen H., Yang B.W., Qian L., Meng Y.S., Bai X.H., Hong X.W., He X., Jiang M.J., Yuan F., Du Q.W. (2022). Deep Learning Prediction of Ovarian Malignancy at US Compared with O-RADS and Expert Assessment. Radiology.

[27] Christiansen F., Epstein E., Smedberg E., Åkerlund M., Smith K., Epstein E. (2021). Ultrasound image analysis using deep neural networks for discriminating between benign and malignant ovarian tumors: Comparison with expert subjective assessment. Ultrasound Obstet. Gynecol. Off. J. Int. Soc. Ultrasound Obstet. Gynecol..

[28] Delle Marchette M., Ceppi L., Andreano A., Bonazzi C., Buda A., Grassi T., Giuliani D., Sina F., Lamanna M., Bianchi T. (2019). Oncologic and fertility impact of surgical approach for borderline ovarian tumours treated with fertility sparing surgery. Eur. J. Cancer.

[29] Timmerman D., Valentin L., Bourne T.H., Collins W.P., Verrelst H., Vergote I. (2000). Terms, definitions and measurements to describe the sonographic features of adnexal tumors: A consensus opinion from the International Ovarian Tumor Analysis (IOTA) Group. Ultrasound Obs. Gynecol..

[30] Timor-Tritsch I.E., Foley C.E., Brandon C., Yoon E., Ciaffarrano J., Monteagudo A., Mittal K., Boyd L. (2019). New sonographic marker of borderline ovarian tumor: Microcystic pattern of papillae and solid components. Ultrasound Obs. Gynecol..

[31] Gupta A., Jha P., Baran T.M., Maturen K.E., Patel-Lippmann K., Zafar H.M., Kamaya A., Antil N., Barroilhet L., Sadowski E. (2022). Ovarian Cancer Detection in Average-Risk Women: Classic- versus Nonclassic-appearing Adnexal Lesions at US. Radiology.

[32] Barnett A.J., Schwartz F.R., Tao C., Chen C., Ren Y., Lo J.Y., Rudin C. (2021). A case-based interpretable deep learning model for classification of mass lesions in digital mammography. Nat. Mach. Intell..

[33] He K., Zhang X., Ren S., Sun J. Deep Residual Learning for Image Recognition. Proceedings of the 2016 IEEE Conference on Computer Vision and Pattern Recognition (CVPR).

[34] Ding X., Zhang X., Zhou Y., Han J., Ding G., Sun J. Scaling Up Your Kernels to 31 × 31: Revisiting Large Kernel Design in CNNs. Proceedings of the 2022 IEEE/CVF Conference on Computer Vision and Pattern Recognition (CVPR).

[35] Ronneberger O., Fischer P., Brox T. (2015). U-Net: Convolutional Networks for Biomedical Image Segmentation. International Conference on Medical Image Computing and Computer-Assisted Intervention.

[36] Bokhovkin A., Burnaev E. (2019). Boundary Loss for Remote Sensing Imagery Semantic Segmentation. International Symposium on Neural Networks.

[37] Loshchilov I., Hutter F. Fixing Weight Decay Regularization in Adam. Proceedings of the Sixth International Conference on Learning Representations.

[38] Sandler M., Howard A., Zhu M., Zhmoginov A., Chen L.C. MobileNetV2: Inverted Residuals and Linear Bottlenecks. Proceedings of the 2018 IEEE/CVF Conference on Computer Vision and Pattern Recognition (CVPR).

[39] Grandini M., Bagli E., Visani G. (2020). Metrics for Multi-Class Classification: An Overview. arXiv.

[40] Jia S., Xiang Y., Yang J., Shi J., Jia C., Leng J. (2020). Oncofertility outcomes after fertility-sparing treatment of bilateral serous borderline ovarian tumors: Results of a large retrospective study. Hum. Reprod..

[41] Alcázar J., Olartecoechea B., Guerriero S., Jurado M. (2013). Expectant management of adnexal masses in selected premenopausal women: A prospective observational study. Ultrasound Obstet. Gynecol. Off. J. Int. Soc. Ultrasound Obstet. Gynecol..

[42] May T., Oza A. (2019). Conservative management of adnexal masses. Lancet Oncol..

[43] Chen L.-C., Papandreou G., Schroff F., Adam H. (2017). Rethinking atrous convolution for semantic image segmentation. arXiv.

[44] Liu Z., Lin Y., Cao Y., Hu H., Wei Y., Zhang Z., Lin S., Guo B. (2021). Swin transformer: Hierarchical vision transformer using shifted windows. arXiv.

