# Supplementary material for: A Deep Learning Model System for Diagnosis and Management of Adnexal Masses
Source: Cancers (Basel). 2022 Oct 27;14(21):5291. doi: 10.3390/cancers14215291 (PMC9659123; doi:10.3390/cancers14215291)
Supplement: Supplementary file 1 [file cancers-14-05291-s001.zip › cancers-1936876-supplementary.pdf]

Supplementary Materials: A Deep Learning Model System for Diagnosis and Management of Adnexal Masses

Jianan Li, Yixin Chen, Minyu Zhang, Peifang Zhang, Kunlun He, Fengqin Yan, Jingbo Li, Hong Xu, Daniel Burkhoff, Yukun Luo, Longxia Wang and Qiuyang Li

Table S1. The comparison of dices among different segmentation models.

Variables	Mass Segmentor			Papillary Segmentor		
	Internal Validation Dataset	External Test Dataset 1	External Test Dataset 2	Internal Validation Dataset	External Test Dataset 1	External Test Dataset 2
deeplabv3 resnet18 [1]	0.921	0.903	0.909	0.827	0.830	0.837
deeplabv3 Resnet50 [1]	0.929	0.907	0.914	0.841	0.842	0.844
Swim Transformer [2]	0.933	0.917	0.897	0.848	0.844	0.837
Unet resnet18 [3]	0.939	0.911	0.905	0.866	0.848	0.858*
Unet-resnet50[3]	0.941	0.921	0.907	0.877*	0.846	0.851
Unet LKResnet-18	0.945*	0.923*	0.912*	0.864	0.852*	0.855

* Optimal dices among six models. LKResnet-18 has the most optimal dices.

References

1. Chen, L.-C.; Papandreou, G.; Schroff, F.; Adam, H. Rethinking atrous convolution for semantic image segmentation. *arXiv* **2017**, arXiv:1706.05587.
2. Liu, Z.; Lin, Y.; Cao, Y.; Hu, H.; Wei, Y.; Zhang, Z.; et al. Swin transformer: Hierarchical vision transformer using shifted windows. *arXiv* **2021**, arXiv:2103.14030.
3. Ronneberger, O.; Fischer, P.; Brox, T. U-net: Convolutional networks for biomedical image segmentation. In Proceedings of the International Conference On Medical Image Computing and Computer-Assisted Intervention, Strasbourg, France, October 2021.
